# Highlights of Model Quality Assessment in CASP16

**DOI:** 10.1002/prot.70035

**Published:** 2025-08-14

**Authors:** Alisia Fadini, Recep Adiyaman, Shaima N. Alhaddad, Behnosh Behzadi, Jianlin Cheng, Xinyue Cui, Nicholas S. Edmunds, Lydia Freddolino, Ahmet G. Genc, Fang Liang, Dong Liu, Jian Liu, Quancheng Liu, Liam J. McGuffin, Pawan Neupane, Chunxiang Peng, David R. Shortle, Meng Sun, Haodong Wang, Qiqige Wuyun, Guijun Zhang, Xuanfeng Zhao, Wei Zheng, Randy J. Read

**Affiliations:** ^1^ Cambridge Institute for Medical Research, University of Cambridge Cambridge UK; ^2^ School of Biological Sciences University of Reading Reading UK; ^3^ Department of Electrical Engineering and Computer Science University of Missouri Columbia Missouri USA; ^4^ College of Information Engineering, Zhejiang University of Technology Hangzhou China; ^5^ Department of Biological Chemistry University of Michigan Ann Arbor Michigan USA; ^6^ Department of Computational Medicine and Bioinformatics University of Michigan Ann Arbor Michigan USA; ^7^ Department of Biological Chemistry Johns Hopkins University School of Medicine Baltimore Maryland USA; ^8^ Department of Computer Science and Engineering Michigan State University East Lansing Michigan USA; ^9^ NITFID, School of Statistics and Data Science, AAIS, LPMC and KLMDASR Nankai University Tianjin China

**Keywords:** computational molecular biology, molecular models, protein conformation, protein domains

## Abstract

Model quality assessment (MQA) remains a critical component of structural bioinformatics for both structure predictors and experimentalists seeking to use predictions for downstream applications. In CASP16, the Evaluation of Model Accuracy (EMA) category featured both global and local quality estimation for multimeric assemblies (QMODE1 and QMODE2), as well as a novel QMODE3 challenge—requiring predictors to identify the best five models from thousands generated by MassiveFold. This paper presents detailed results from several leading CASP16 EMA methods, highlighting the strengths and limitations of the approaches.

## 1 Introduction

In CASP16, the Evaluation of Model Accuracy (EMA) category assessed predictors' capabilities to estimate the quality of models, or perform model quality assessment (MQA). Accurate model quality assessment is essential not only for predictors and ranking pipelines but also for downstream users seeking to interpret predicted structures or experimentally validate hypotheses inspired by them. A particular challenge in this context is the ability to evaluate model confidence for multimeric assemblies.

Building on CASP15, the CASP16 EMA experiment further expanded the evaluation. It included the established QMODE1 and QMODE2 tasks, which focus on global and local accuracy of complex models, as well as a new QMODE3 task: selecting the five best models from a pool of thousands generated by the MassiveFold [1] tool. This addition enabled benchmarking under conditions of high model pool redundancy and highlighted the usefulness of refined scoring combinations.

This paper presents detailed results and insights from top‐performing EMA predictor groups in the EMA category of CASP16. Because the underlying software tools frequently have either “EMA” or “MQA” in their names, we use the two terms more or less interchangeably below. The highlights presented are framed within the context of the accompanying “Model Quality Assessment for CASP16” paper [2], with the aim to provide a more granular understanding of what went right, what went wrong, and what was learned in this year's EMA category.

As structure prediction methods have advanced, older quality metrics have been surpassed, and new evaluation criteria have been developed to probe more advanced challenges such as the prediction of assemblies rather than just monomers or domains. In CASP16, attention was focused on the existing QMODE1 and QMODE2 subcategories, as well as a new QMODE3 criterion. Because these names are somewhat obscure, these evaluation criteria are briefly summarized here.

QMODE1 focuses on global quality metrics for predicted structures of assemblies. This is divided into *SCORE*, which evaluates the overall structural topology from a global superposition; and *QSCORE*, which focuses on the accuracy of the predicted interfaces between components of the assembly.

QMODE2 focuses on local evaluation of interface prediction, looking at both the accuracy of the local environment of residues in the interface, as well as on the identification of which residues contribute to interfaces.

QMODE3 was developed in response to the success of the MassiveFold large‐scale model generation tool in CASP15 [1], in which the challenge is to pick the best one of many models. QMODE3 was scored by evaluating how well the model assessment groups did in choosing the best 5 models from up to 8040 MassiveFold models.

## 2 Results

Five groups (described in Table 1) were invited to contribute to this highlights paper. The performance of these groups in the various assessment subcategories of the CASP16 EMA category is summarized in Table 2. Groups were chosen on the basis of their performance in individual or multiple subcategories, as well as to illustrate diverse approaches to the tasks. These approaches can be categorized in terms of single‐model methods, where no comparisons are done with other models, quasi‐single‐model methods, where the assessment algorithm generates its own models for comparison, and consensus methods, where individual models are judged by their consensus with the other models. Table 2 assigns the methods to these categories. As seen in previous editions of CASP, consensus methods generally perform most strongly, followed by quasi‐single‐model methods and then single‐model methods.

**TABLE 1:** Groups contributing to the discussion of highlights for CASP16 EMA.

Group	Members	Sub‐groups (abbreviation)
MIEnsembles‐Server	Chunxiang Peng, Wei Zheng, Qiqige Wuyun, Quancheng Liu, Lydia Freddolino	MIEnsembles‐Server
ModFOLDdock2	Liam J. McGuffin, Nicholas S. Edmunds, Behnosh Behzadi, Shaima N. Alhaddad, Ahmet G. Genc, Recep Adiyaman	ModFOLDdock2 (dock2) ModFOLDdock2R (dock2R) ModFOLDdock2S (dock2S)
MULTICOM	Jian Liu, Pawan Neupane, Jianlin Cheng	MULTICOM MULTICOM_AI (AI) MULTICOM_GATE (GATE) MULTICOM_LLM (LLM) MULTICOM_human (human)
GuijunLab	Guijun Zhang, Dong Liu, Xuanfeng Zhao, Haodong Wang, Fang Liang, Meng Sun, Xinyue Cui	GuijunLab‐Complex (Complex) GuijunLab‐Human (Human) GuijunLab‐PAthreader (PAthreader) GuijunLab‐QA (QA) GuijunLab‐Assembly (Assembly)
SHORTLE	David R. Shortle	SHORTLE

**TABLE 2:** Summary of evaluation criteria, prediction groups and rankings.

Criteria	Prediction groups				
	MIEnsembles‐Server	ModFOLDdock2	MULTICOM	GuijunLab	SHORTLE
QMODE1: SCORE	1: MIEnsembles‐Server (q)	3: dock2 (c) 8: dock2R (c) 14: dock2S (q)	2: LLM (c*) 4: GATE (c) 11: MULTICOM (c) 15: human (c) 22: AI (s)	5: QA (c) 6: Human (c) 9: Assembly (s) 10: PAthreader (s) 17: Complex (s)	—
QMODE1: QSCORE	2: MIEnsembles‐Server (q)	1: dock2 (c) 5: dock2R (c) 9: dock2S (q)	6: LLM (c*) 7: GATE (c) 15: MULTICOM (c) 21: human (c) 25: AI (s)	3: QA (c) 5: Human (c) 8: Assembly (s) 12: Complex (s) 13: PAthreader (s)	—
QMODE2: Interface accuracy	—	1: dock2 (c) 2: dock2R (c) 5: dock2S (q)	—	3: QA (c) 4: Human (c) 6: PAthreader (s) 7: Assembly (s) 8: Complex (s)	—
QMODE2: Interface identity	—	2: dock2S (q) 4: dock2R (c) 5: dock2 (c)	—	3: QA (c) 6: Human (c) 8: PAthreader (s) 9: Complex (s) 10: Assembly (s)	—
QMODE3: monomer	3: MIEnsembles‐Server (q)	—	6: MULTICOM (s*) 7: human (c) 9: GATE (c) 10: LLM (c*) 16: AI (s)	11: Assembly (s) 12: Complex (s) 14: QA (c) 17: PAthreader (s) 18: Human (c,q)	5: SHORTLE (s)
QMODE3: homo‐oligomer	13: MIEnsembles‐Server (q)	—	1: LLM (c*) 2: human (c) 6: GATE (c) 7: MULTICOM (s) 8: AI (s)	3: PAthreader (s) 10: Complex (s) 11: QA (c) 14: Assembly (s) 15: Human (c,q)	5: SHORTLE (s)
QMODE3: hetero‐oligomer	4: MIEnsembles‐Server (q)	—	13: GATE (c) 15: MULTICOM (s) 18: human (c) 21: LLM (c*) 22: AI (s)	2: Human (c,q) 3: Assembly (s) 5: Complex (s) 8: QA (c) 12: PAthreader (s)	7: SHORTLE (s)

*Note*: Entries in the table show the final rank (from the accompanying “Model Quality Assessment for CASP16” paper and the abbreviated name from Table 1 of the sub‐group methods for each prediction group). The letter in parentheses after the method name specifies the class of assessment algorithm: s = single‐model, q = quasi‐single‐model, c = consensus. Methods marked with asterisks use hybrid approaches as noted below^a^ ^,^ ^b^.

^a^ c*: MULTICOM_LLM applied a single‐model approach (EnQA) for targets T1201o, T1207, H1202, and H1204; a consensus approach (PSS) for the remaining targets in QMODE1 and QMODE3.

^b^ s*: MULTICOM used a consensus approach for T1207 and a single‐model approach (EnQA) for all other targets in QMODE3.

It has been noted that consensus methods are less relevant for real‐life use cases than for CASP challenges, because there are rarely many independent models available that have been generated using a variety of algorithms [3]. It is reassuring, then, that quasi‐single‐model methods can be reasonably competitive [4]. It might seem counterintuitive that generating your own reference models adds value to single‐model methods, but the process of generating a model integrates all the information that might have been used for evaluation, and consistency among multiple models will give some indication of reliability.

### 2.1 MIEnsembles‐Server Methods

During CASP16, the MIEnsembles‐Server employed the StrMQA method to evaluate the global modeling quality (SCORE) and interface modeling quality (QSCORE) for protein complexes in QMODE1. It also facilitated the selection of the most accurate protein monomer and complex models from MassiveFold in QMODE3. StrMQA is a machine learning‐based approach that ranks candidate models by measuring their structural similarity to a diverse set of high‐quality reference models generated by DMFold [5, 6], augmented by template information and individual quality assessment (QA) scores from third‐party tools as features.

In general, StrMQA incorporates four types of features: (1) structural similarity scores between candidate models and reference models predicted by DMFold [5, 6], including TM‐score [7, 8], LDDT [9], and DockQ [10], with only the first two applied to protein monomers; (2) structural similarity scores derived from the best templates for biological assemblies in protein complexes or from the best monomer templates in the monomer PDB library; (3) predicted average pLDDT from DeepAccNet [11]; and (4) predicted CAD‐scores from VoroIF‐GNN [12]. For protein complex models, these four types of features are input into two separate random forest regression models to predict the global topology accuracy (predicted TM‐score, pTM) and interface accuracy (pDockQ) of the input models. For protein monomer models, only the first three types of features are used in a single random forest regression model to predict the global topology accuracy (pTM). In StrMQA, we have found that the structural similarity scores with reference predicted structures are particularly critical.

#### 2.1.1 Generation of Predicted Reference Structure‐Based Features

DMFold is utilized to generate high‐quality predicted reference models for StrMQA. The initial step in DMFold involves the creation of multiple sequence alignments (MSAs) for a protein monomer or the monomer component of a protein complex using DeepMSA2 [6], which comprises three sub‐pipelines: dMSA [13], qMSA, and mMSA. These sub‐pipelines are iteratively employed to gather homologous sequences from genomic and metagenomic databases, including Uniclust30 [14], UniRef90 [15], Metaclust [16], Mgnify [17], BFD [16], and the IMG/M [18] large‐scale metagenomics database. The MSAs produced by these sub‐pipelines are subsequently input into AlphaFold2 to predict a set of structural decoys. These decoys are ranked according to their associated pLDDT scores. To ensure both diversity and consensus, the top five ranked MSAs are either directly used in protein monomer modeling or paired as multimer MSAs for protein complex modeling. The second step involves the generation of structural decoys using a modified AlphaFold2 modeling engine. For each MSA, 100 decoys are generated and ranked based on the pLDDT score for monomer targets or confidence scores (0.8ipTM + 0.2pTM) for complex targets. Finally, five top‐ranked DMFold decoys from different MSAs are selected as the final reference models. The structural similarity between each reference model from DMFold and the input models of the QA target is then calculated, yielding five TM‐scores, five DockQ scores, and five LDDT scores for each model of the given QA target.

#### 2.1.2 Generation of Template‐Based Features

A hybrid template detection protocol is employed to collect structural templates. For protein monomer targets, Foldseek/US‐align [19, 20, 21] is used to identify the best templates from a non‐redundant PDB library, using the input model of the QA target as the query structure. For protein complex targets, Foldseek [20] is initially applied to rapidly search for candidate monomer templates corresponding to the component monomer chains. Subsequently, biological assemblies, which include at least two templates from different chains, are gathered into an assembly template pool. Finally, US‐align [19] is used to select the best biological assembly based on structural similarity to the query model. The TM‐score of an input structure to the best assembly is then utilized as a template‐based feature for the StrMQA method.

MIEnsembles‐Server used the same method to select QMODE3 as for QMODE1 and just selected the top 5 decoys using a global score.

DMFold, DeepMSA2, TM‐score and US‐align are freely available at https://seq2fun.dcmb.med.umich.edu/DMFold/. StrMQA is still in development.

#### 2.1.3 MIEnsembles‐Server Summary

**FIGURE 1:**
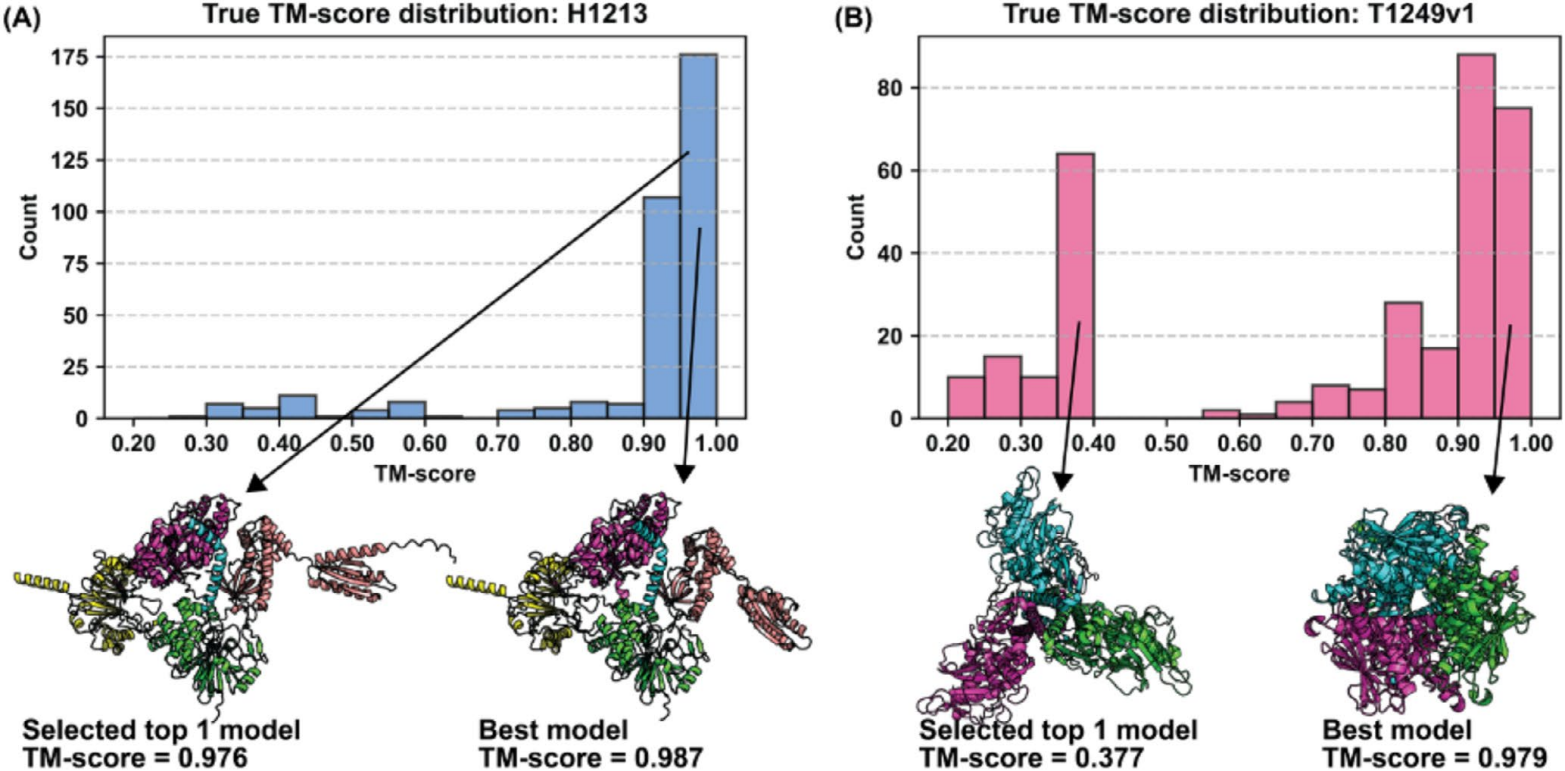
MIEnsembles‐Server: What went right and what went wrong? (A) Performance on target H1213, where StrMQA succeeded in choosing a good model. (B) Performance on target T1249v1, where StrMQA chose reference models in the wrong conformation. See text for further details.

What went right? The MIEnsembles‐Server (StrMQA) achieved first place in overall folding accuracy scoring (QMODE1‐SCORE) and second place in overall interface accuracy scoring (QMODE1‐QSCORE). The strong performance of StrMQA can be largely attributed to the high‐quality models generated by DMFold. Although StrMQA can be considered a consensus‐based approach to some extent, it diverges from traditional consensus‐based methods by not performing mutual scoring or evaluation among the models within the model pool. This distinction arises because conventional consensus‐based methods heavily rely on the number of high‐quality models within the pool, which often vary in quality and are not directly controlled by the assessment system itself. In contrast, the models predicted by DMFold are selected as reference models, with their structural similarity to the candidate models of QA target being evaluated. To mitigate excessive dependence on DMFold predicted structures, additional information and features are incorporated to complement the data provided by DMFold. The high accuracy of these reference models is a crucial factor contributing to StrMQA's strong performance in the EMA category. Figure 1A presents an example of StrMQA's performance for target H1213. For this target, the five reference models generated by DMFold had TM‐scores compared to the experimental target around 0.95, with four exceeding 0.97 and one reaching 0.94. The availability of these high‐quality reference models enabled StrMQA to achieve superior performance. Consequently, the top‐ranked model selected by StrMQA attained a TM‐score of 0.976, with a difference of only 0.011 from the best model. Overall, for this target, StrMQA demonstrated a TM‐score loss of 0.01 and an oligo‐GDTTS loss of 0.05. The Pearson correlation coefficient (PCC) between the true TM‐score and the predicted overall folding accuracy was 0.99. StrMQA is most successful for monomer assessment because it relies on newly generated structural models for quality assessment. In other words, the quality of the reference model directly impacts the performance of our quality assessment (QA) method, and it is substantially easier for our pipeline to provide high‐quality monomer structure predictions. The case study described for H1213 (described above) also illustrates this point.

What went wrong? Figure 1B illustrates an example of a poor model for T1249v1, which was incorrectly ranked at the top by StrMQA. This target exhibits two alternative conformations, and DMFold successfully predicted these two alternative structural conformations among all generated decoys. However, due to an inherent weakness in the default AlphaFold2 model ranking, the correctly predicted structure for T1249v1 was assigned a lower confidence score. As a result, none of the top five reference models accurately represented the topology of T1249v1. The TM‐scores of these five reference models, when compared to the experimental structure of T1249v1, were all below 0.40. Furthermore, additional information could not compensate for the detrimental impact of the incorrect reference models on this target. This is evidenced by a PCC of 0.52 between the TM‐score and pLDDT from DeepAccNet, and a negative PCC of −0.31 between the TM‐score and CAD‐score from VoroIF‐GNN. Consequently, StrMQA's performance on this target was suboptimal, as the PCC between the true TM‐score and the predicted overall folding accuracy was negative. Despite the presence of several high‐quality models among all submissions, StrMQA failed to identify them. The TM‐score of the top‐ranked model selected by StrMQA was 0.377, whereas the TM‐score of the best model was 0.979.

Assessors raised the question of whether a reason why mean pLDDT would not work as well as a criterion for selecting multimer MSAs could be difficulties of sequence linking when sequence annotations are limited (the issue is described in the original DMFold publication). This could indeed be one of the reasons. However, another contributing factor is that the confidence score used by DMFold (0.8ipTM + 0.2pTM, the same as AlphaFold2 for protein complex modeling) is sometimes not sufficiently sensitive to accurately identify the correct models in complex modeling. If an incorrect DMFold model is selected as a reference model, it will lead to an inaccurate assessment of the decoys submitted by CASP groups. However, as we noted in the previous discussion, the monomer modeling ranking score (pLDDT) appears to be more robust for selecting high‐quality reference models for monomer proteins than 0.8ipTM + 0.2pTM for protein complexes. An example of this is T1249v1. Although we had an excellent model in the MIEnsembles‐Server modeling pool, its confidence score was low, resulting in its exclusion from the top five models. Consequently, the incorrect top five models were used as reference models for StrMQA, leading to erroneous quality assessment results.

### 2.2 ModFOLDdock2 Methods

We developed three distinct variants of ModFOLDdock2 for our automated QMODE2 submissions, each optimized for performance on different metrics. Firstly, we developed the standard ModFOLDdock2 variant with global scores optimized for positive linear correlations with the observed scores. Secondly, we developed ModFOLDdock2R with global scores optimized for ranking, where the top‐ranked models would have a higher overall observed accuracy. Finally, we developed ModFOLDdock2S, a quasi‐single model approach to score models, which integrated our new version of MultiFOLD [22] to generate reference sets of multimer models. Our QMODE3 manual submissions were made using two methods: ModFOLD9Q, a quick version of ModFOLD9 [23] for monomer ranking, and ModFOLDdock2Q, a quick version of ModFOLDdock2R for multimer ranking.

The primary differences from the original version of ModFOLDdock [24] included the integration of several new component scores and optimization using new target functions that were based on assessors' scores from CASP15. Each server variant integrated specific combinations of component scoring methods. We developed nine different consensus methods, which carried out all‐against‐all comparisons of submitted models using different scores: QS‐bestJury, DockQ‐waveJury, TM‐scoreJury, Oligo‐GDTJury, lDDTJury, CADJury, PatchQSJury, PatchDockQJury, and ModFOLDIA. We used OpenStructure [25] version 2.7 to obtain the QS [26], DockQ [27], TM‐score [8], GDT [8], lDDT [9] and CAD [28] scores for each pairwise comparison (using the “ost compare‐structures” action). We also used three different single‐model scoring methods: VoroIF [12], VoroMQA [29], and CDA [30, 31]. The VoroIF(VoroIF‐GNN) and VoroMQA(voronota‐js‐voromqa) methods were used off‐the‐shelf, VoroIF with the “as‐assembly true” and “local‐column true” options, and VoroMQA with the “inter‐chain” and “output‐dark‐scores” options. Our Contact Distance Agreement (CDA) score used the contact prediction profiles that resulted from generating multimer models with LocalColabFold [32].

**FIGURE 2:**
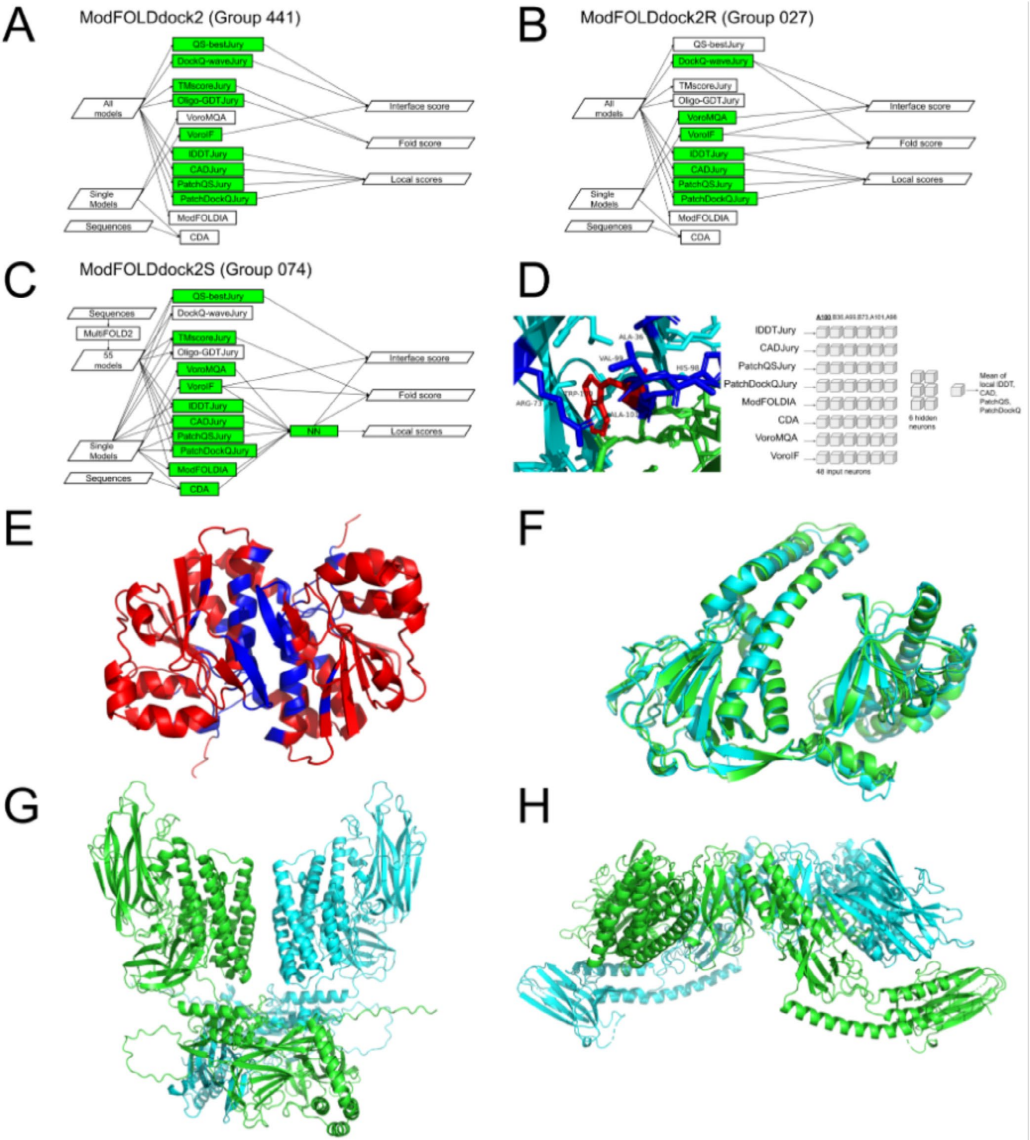
The ModFOLDdock2 methods, what went right and what went wrong. (A) Flow of data and processes for ModFOLDdock2 with scores optimized for global correlations. The 3D models and the target sequences are inputs (left), which are then processed by the specific component methods highlighted in green (middle) to produce each of the output scores required for QMODE2 submissions (right). (B) Flowchart for ModFOLDdock2R optimized for global ranking or model selection. (C) Flowchart for ModFOLDdock2S, a quasi‐single model approach using MultiFOLD2. (D) The multi‐layer perceptron (MLP) for the ModFOLDdock2S local scores with an example of the scoring. Left: Scoring an example residue (red sticks, TRP‐100 on chain *A* = A100) includes the scores for the five closest contacting interface residues (≤ 8 Å) in order of their proximity to A100 (blue sticks). Right: NN architecture. 48 input neurons (8 scores × 6 residues), 6 hidden neurons, and 1 output (mean of the local lDDT, CAD, PatchQS, and PatchDockQ scores). (E) Example of excellent QMODE1/2 performance by ModFOLDdock2. The top selected model for T1292o is colored by predicted interface residue accuracy from blue (high confidence of interface residue) to red (non‐interface residue or very low confidence). QS Loss = 0.005 and QS AUC = 0.9987. (F) Example of excellent QMODE3 performance by ModFOLD9Q with zero penalty overall. The top selected model for T1212 (cyan) superposed with the native structure (green). Penalty_w = 0.000 (G) Example of poor QMODE1/2 performance by ModFOLDdock2. The top selected T1218o model, colored by chain identifiers. (H) The T1218o native structure, colored by chain identifiers.

Figure 2A–C shows flow charts for the three variants of the ModFOLDdock2 methods, which we used for our QMODE2 submissions. The component scoring methods contributing to the final output scores for each ModFOLDdock2 variant are highlighted in green, and the arrows indicate how they contribute to each output score. The ModFOLDdock2 variant (Figure 2A) produced predicted scores optimized for positive linear correlations with the observed scores, that is, the predicted quality scores correlated well with the observed quality scores, according to the assessors' formulae for CASP15 multimer models [33]. The ModFOLDdock2R variant (Figure 2B) used different combinations of methods to contribute to the final global scores, which were optimized for global ranking or model selection. The ModFOLDdock2S variant (Figure 2C) also used different components for the global scores. Furthermore, the local scores were fed into a neural network trained to learn the mean of the observed local interface scores (Figure 2D). Importantly, the ModFOLDdock2S variant also used a quasi‐single model approach, relying on the generation of reference sets of models using MultiFOLD2.

For the QMODE3 category, we developed quicker versions of our ModFOLD9 (ModFOLD9Q) and ModFOLDdock2 (ModFOLDdock2Q) methods to manually score and rank the monomeric and multimeric MassiveFold models, respectively. In the ModFOLD9Q method, the top 40 ModFOLD9 ranked server models for Phase 1 targets were used as reference sets for comparison against the MassiveFold models using the mean of the GDTJury and lDDTJury scores. For multimers, we developed ModFOLDdock2Q, where up to 40 MassiveFold models were first selected using VoroIF and then used as reference sets for comparison against the MassiveFold models, which were scored in the same way as ModFOLDdock2R.

The ModFOLDdock2 server is available at: https://www.reading.ac.uk/bioinf/ModFOLDdock/. ModFOLDdock2 is also available to download via the MultiFOLD2 docker image: https://hub.docker.com/r/mcguffin/multifold2.

#### 2.2.1 ModFOLDdock2 Summary

What went right? The ModFOLDdock2 method ranked first place for both the global and local interface accuracy scoring (QMODE1‐QSCORE and QMODE2). The other ModFOLDdock2 variants were placed among the top five methods according to many other metrics. The ModFOLDdock2 variant performed well according to correlations and ROC scores, whereas the ModFOLDdock2R performed better according to the loss than the other variants (https://predictioncenter.org/casp16/results.cgi?tr_type=accuracy), which was expected due to their specific score optimizations.

Figure 2E shows an example of excellent ModFOLDdock2 performance according to the QS score. For this target, ModFOLDdock2 achieved near‐perfect loss and ROC scores and very strong correlations. The McGuffin group also ranked in second place in the QMODE3 category on monomers. This indicated that our ModFOLD9Q method reliably selected reference sets of models for accurately ranking the top monomer models (Figure 2F).

What went wrong? Despite these successes, specific tasks remained challenging. Figure 2G shows an example of a model for T1218o, which was incorrectly ranked at the top by ModFOLDdock2. The native conformation is shown in Figure 2H. For this target, models with incorrect conformations were selected and ranked at the top. This led to negative correlations, random ROC scores, and a large loss. We also had issues with our QMODE3 predictions for multimers. We relied on VoroIF for the rapid selection of reference models; however, for several targets, the top‐selected models had severe clashes, which greatly impacted our overall performance in that category. Indeed, these different approaches used for monomers and multimers help explain why our method performed much better for monomer targets than for multimer targets in QMODE3. For ranking the MassiveFold monomers, we relied on our trusted ModFOLD9 method for selecting models. ModFOLD9 is very reliable for scoring monomer models (it currently outperforms all other methods in CAMEO), but it cannot be used for multimer models. For multimers, we needed a rapid scoring method to identify reference sets of models, so we chose VoroIF for this part because it performed very well in CASP15 and was relatively quick to deploy. We therefore learned that, unfortunately, VoroIF could not be relied upon to identify the errors for some MassiveFold models in cases where the subunits had significant clashes or overlaps. This caused errors with our subsequent model comparisons due to the low‐quality sets of reference models. In future, we will use ModFOLDdock2R to select from the Phase 1 models to be used as reference sets. In addition, our reliance on manual submission for QMODE3 led to human errors—we missed a few submission deadlines and could not inspect the models before submission due to lack of time. This could be resolved through automation.

A first point noted by assessors was the performance difference between ModFOLDdock2 and ModFOLDdock2R in the QMODE1 category. ModFOLDdock2 achieved stronger results because its predicted scores more accurately reflected the absolute observed model quality scores, so the method performs better according to the Pearson, Spearman, and ROC scores, which account for 3/4 of the scores making up the final method ranking. ModFOLDdock2R does better according to the loss, which is not unexpected as it is optimized to rank the best models at the top. However, because the relationships between the observed and predicted scores are not linear, ModFOLDdock2R performs worse on the correlations and ROC scores.

A second point noted by the assessors was that, in CASP15, the QSCORE was significantly lower than the baseline assembly consensus (AC) measure; while those positions were reversed in CASP16. We can understand this by considering that, in CASP16, we optimized for more appropriate target functions and included more input scores that better reflect the QSCOREs. Furthermore, our handling of larger structures also improved; we were more efficient at chain mapping, and we had faster machines and faster methods for scoring.

### 2.3 MULTICOM EMA Methods

During CASP16, three predictors of the MULTICOM group participated in two modes of the EMA category: QMODE1 for global model quality estimation and QMODE3 for selecting the top five models and achieved competitive performance.

In QMODE1, MULTICOM_LLM used a single model EMA method (EnQA [34]) based on a 3D‐equivariant neural network to predict global fold accuracy of the models for three early targets (i.e., T1201o, H1202, and H1204) and then switched to use the average pairwise similarity score (PSS) [35] between a model and other models, calculated by MMalign [36] (a multi‐model consensus method), to estimate model accuracy for the remaining targets. MULTICOM_GATE utilized a graph transformer (GATE [37]) integrating the quality features of individual models and the similarity between models to predict global fold accuracy (e.g., TM‐score [7, 21]).

In QMODE3, to reduce the time complexity of selecting the top five models from many MassiveFold [1] generated AlphaFold2 models (usually thousands), 200 models were first selected using AlphaFold‐Multimer's confidence scores for two predictors MULTICOM_LLM and MULTICOM_human to make the final selection, respectively. MULTICOM_LLM used here is the same as the one used in QMODE1. MULTICOM_human used the average of three complementary quality scores: the average CAD‐score [28] between a model and other models, the quality score predicted by a geometry‐complete neural network (GCPNet‐EMA [38]), and the quality score predicted by a variant of the GATE model trained on our in‐house CASP15 complex models generated by MULTICOM3 [39, 40], to select five models. This GATE variant uses AlphaFold‐Multimer model features, such as confidence score, ipTM, and pTM, inter‐chain predicted aligned errors (< 5 Å), and mpDockQ score [41] as well as the features of the default GATE [37] to predict the quality of structural models. If a complex target was too large to obtain AlphaFold‐Multimer features for its models, the default GATE [37] without the features was used to generate quality scores for them.

The source code of the methods used by the MULTICOM group for the EMA category is available at https://github.com/BioinfoMachineLearning/gate, with running instructions provided at https://github.com/BioinfoMachineLearning/gate/tree/main/MULTICOM_EMA.

#### 2.3.1 MULTICOM EMA Summary

**FIGURE 3:**
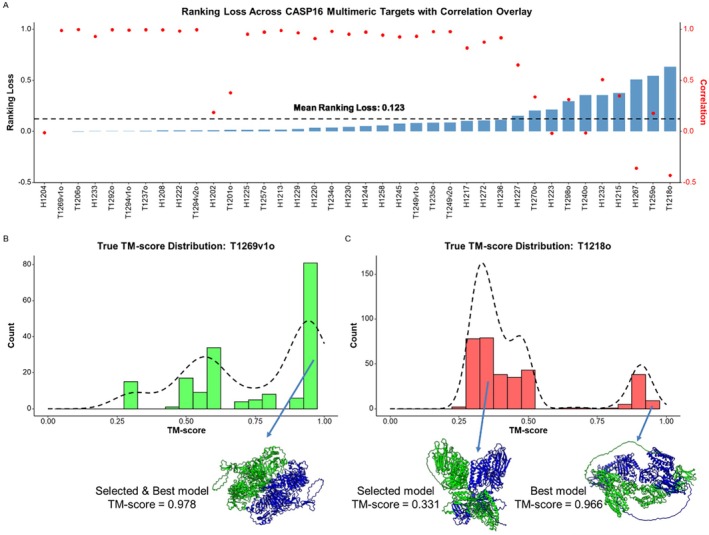
(A) Per‐target ranking loss and Pearson's correlation of MULTICOM_LLM in QMODE1. Red dots denote correlations and blue bars ranking losses. (B) The true TM‐score distribution of the models of T1269v1o. (C) The true TM‐score distribution of the models of T1218o.

The MULTICOM group delivered competitive performance in QMODE1, with MULTICOM_LLM ranking second and MULTICOM_GATE fourth in QMODE1 for the global fold accuracy estimation. Figure 3A illustrates the per‐target Pearson's correlation and ranking loss for MULTICOM_LLM across multimeric targets. On average, MULTICOM_LLM achieved a ranking loss of 0.123 and a Pearson's correlation of 0.686. Notably, 25 out of 38 targets (65.79%) have a correlation higher than 0.686, while 28 targets (73.68%) have a ranking loss lower than 0.123. Generally, the targets with a high correlation tend to have a low loss, except for the early targets (T1201o, H1202, and H1204) to which EnQA was applied EnQA, a single‐model quality assessment method, performed well in ranking good models at the top for these targets but failed to approximate predicted quality scores relative to native scores, leading to low correlation. For instance, for H1204 (a very hard nanobody target), EnQA successfully identified the best model as top 1 with a ranking loss of 0 but had almost zero Pearson's correlation of −0.01. In contrast, other multi‐model quality assessment methods such as MULTICOM_GATE failed to rank a good model at the top for this target and had a high loss (0.284).

The PSS method used in MULTICOM_LLM performed well when the model pool contained a large cluster of good‐quality structures. For instance, for T1269v1o (Figure 3B), where 58% of models had TM‐scores > 0.7, PSS reliably identified the best model (TM‐score = 0.978), resulting in a ranking loss of 0. However, its performance declined in cases where the similar low‐quality models formed the largest cluster, such as T1218o (Figure 3C). In these cases, PSS prioritized the low‐quality models in the largest cluster because they have higher PSS scores, underscoring a key limitation of the consensus‐based scoring for model pools with few good models and many similar bad models. The ranking loss of PSS for T1218o is 0.635.

MULTICOM_GATE tackled this issue by constructing pairwise similarity graphs between models, sampling subgraphs evenly from model clusters, and utilizing graph transformers to predict model quality. This enabled it to outperform PSS in some cases, such as the nanobody target H1215, where it successfully identified a good model (loss: 0.003) from a smaller, higher‐quality cluster that PSS in MULTICOM_LLM overlooked, leading to a high loss of 0.377. However, the average loss of MULTICOM_GATE and MULTICOM_LLM is still comparable across all the targets (0.122 vs. 0.123).

It is worth noting MULTICOM_LLM and MULTICOM_GATE treated AlphaFold2‐ and AlphaFold3‐generated models in model pools equally. MULTICOM_LLM ranked an AlphaFold3 model at top 1 for 13 out of 38 targets (34.2%); while MULTICOM_GATE ranked an AlphaFold3 model at top 1 for 11 out of 38 targets (28.9%). Here, we distinguish AlphaFold3 models from AlphaFold2 models according to the plDDT values at the atom level because the former has different plDDT values for different atoms in the same residue, while the latter has the same plDDT values for all the atoms of the same residue.

In QMODE3, MULTICOM_LLM, and MULTICOM_human ranked first and second for homo‐multimers among CASP16 EMA predictors, based on their weighted penalty scores across global, local, and interface quality metrics. Here, the penalty score is calculated as the sum of the mean square error (MSE) between the true quality scores of the top 5 models selected by a method and those of the actual top 5 models (ranked by their true quality scores). Lower penalty scores indicate better model selection accuracy, while higher scores reflect greater mismatches. Both MULTICOM_LLM and MULTICOM_human also outperformed the default AlphaFold2‐Multimer's confidence score in selecting models for homo‐multimers. However, they struggled with hetero‐multimers, particularly having higher penalty scores in interface quality metrics.

The reason may be that MULTICOM_LLM (or MULTICOM_human) used the average TM‐score (or CAD‐score) between a model and other models to select the top five models, which mainly considered global fold accuracy without directly taking the interface quality into account.

### 2.4 GuijunLab Methods

**FIGURE 4:**
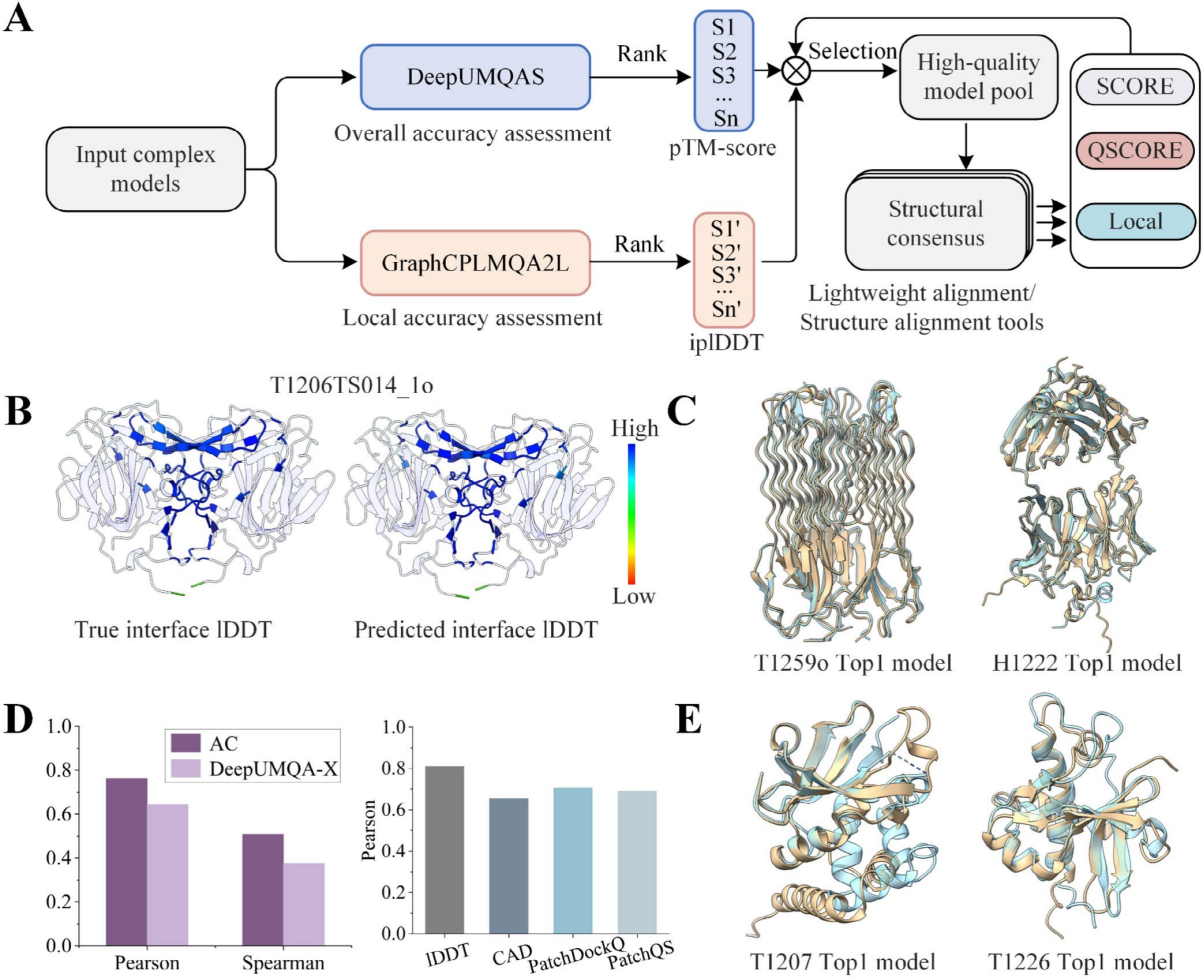
The pipeline of DeepUMQA‐X method: What went right and what went wrong. (A) DeepUMQA‐X evaluates protein model accuracy based on two single‐model methods, DeepUMQAS and GraphCPLMQA2L, with a consensus strategy. (B) True local interface accuracy (left panel) and predicted local interface accuracy of DeepUMQA‐X (right panel) for the CASP16 model T1206TS014_1o in lDDT metric. Red represents low quality regions, and blue represents high quality regions. (C) Top‐ranked MassiveFold models selected by DeepUMQA‐X for CASP16 complex targets T1259o and H1222. The top‐ranked model for T1259o (left panel) has QS‐best [26] = 0.961 and TM‐score = 0.987, and the top‐ranked model for H1222 (right panel) has QS‐best = 0.758 and TM‐score = 0.961. The yellow regions represent the top‐ranked model, and light blue regions represent native structure. (D) Pearson and Spearman performance comparison of official assembly consensus (AC) and DeepUMQA‐X for global accuracy assessment using Oligo‐GDTTS for all CASP16 models (left panel). Pearson performance comparison of DeepUMQA‐X for local interface accuracy assessment based on lDDT, CAD, PatchDockQ and PathQS metrics, across all CASP16 models (right panel). (E) Top‐ranked MassiveFold models selected by DeepUMQA‐X for CASP16 monomer targets T1207 and T1226. The top‐ranked model for T1207 (left panel) has lDDT = 0.515 and TM‐score = 0.684, and the top‐ranked model for T1226 (right panel) has lDDT = 0.535 and TM‐score = 0.753. The yellow regions represent the top‐ranked models, and the light blue regions represent the native structures.

In CASP16, we significantly advanced our previously established EMA methods [42, 43, 44, 45, 46, 47] by developing two distinct single‐model approaches: GraphCPLMQA2L (Group: GuijunLab‐PAthreader) for model local accuracy estimation and DeepUMQAS (Group: GuijunLab‐Complex) for model global accuracy estimation. Building on these developments, we further incorporated a consensus‐based strategy to establish DeepUMQA‐X (Group: GuijunLab‐QA & Human), a unified framework for scoring, ranking, and selecting complex protein models, as illustrated in Figure 4A.

#### 2.4.1 Single‐Model Method for Local Accuracy Estimation

GraphCPLMQA2L is an enhanced version of our previous single‐model method, GraphCPLMQA [42]. This approach employs a graph‐coupled network to integrate sequence, structural, evolutionary, and statistical features, facilitating the accurate characterization of relationships between individual residues and their corresponding residue‐level accuracy, as quantified by plDDT [9]. Specifically, the sequence features include one‐hot encoding, relative position encoding, and the physicochemical properties of amino acids; the structural features include triangular position, voxelization, residue‐residue distance and orientation maps, backbone torsion angles and bond lengths, along with secondary structure information; the evolutionary features include embeddings from the protein language model ESM [48] and AlphaFold Evoformer [49]; the statistical features [11] include Rosetta energy terms and Blosum62 scores. Given a protein model, these features are first extracted and input into a deep graph network module to predict a reference model, which serves as a geometric constraint approximating the native structure. Subsequently, the geometric constraint, along with the extracted features, is employed in a deep convolutional neural network leveraging transformer‐based strategies to predict distance bias and contact maps. Finally, the local residue accuracy score (plDDT) is computed based on the predicted maps.

#### 2.4.2 Single‐Model Method for Global Accuracy Estimation

DeepUMQAS extends DeepUMQA [43] for global accuracy estimation (i.e., SCORE), as quantified by TM‐score [8]. Similarly, DeepUMQAS also extracts sequence, structural, and statistical features from protein models. These features are divided into three hierarchical levels according to the relationship between the residues and their surrounding environment: residue‐microenvironment, residue‐macroenvironment, and global residue representations. These representations are processed in parallel through hierarchical network architecture to predict the global quality score, pTM‐score. The network architecture integrates a convolutional neural network, a transformer network, and a graph attention network, leveraging their complementary strengths to enhance prediction accuracy. Notably, the key feature of this method is its exclusive reliance on the intrinsic information of the model structure itself, deliberately omitting any incorporation of evolutionary information. This design ensures that the prediction results remain entirely independent of the MSAs or template‐based information (i.e., evolutionary information) typically utilized in protein structure modeling, thereby offering a new and fully decoupled solution for protein model accuracy estimation.

#### 2.4.3 DeepUMQA‐X Framework

DeepUMQA‐X integrates two independent single‐model methods, GraphCPLMQA2L and DeepUMQAS, with a consensus strategy to establish a comprehensive framework for scoring, ranking, and selecting protein models (Figure 4A). Specifically, given a set of protein models, the single‐model methods GraphCPLMQA2L and DeepUMQAS are first used to evaluate residue‐level local quality and topology‐level global quality, respectively. The models are then ranked based on their local and global quality scores to jointly select high‐quality candidate structures. The selection criteria for candidate models are as follows: (a) The average local interface residue quality score (iplDDT) must rank within the top *n*% of models (GuijunLab‐QA: *n* = 25; GuijunLab‐Human: *n* = 50); (b) The global quality score (pTM‐score) must rank within the top *m*% of models (GuijunLab‐QA: *m* = 25; GuijunLab‐Human: *m* = 50); (c) The stoichiometry must be correct; (d) The maximum residue gap is 10%; (e) Each single‐chain structure must contain interface residues. The candidate structures are then used as reference models to align with all model structures through the protein structure alignment suite, OpenStructure [25], calculating the similarity scores of each model in terms of overall, interface, and local metrics. Finally, based on these scores, the models are reselected and reassessed to refine the evaluation quality of distinct metrics. It is worth noting that we introduced a lightweight interface alignment strategy specifically tailored to improve the efficiency of structure alignment for large assemblies and massive models. This method calculates alignment scores by utilizing sequence consensus among interchain interface residues, thereby substantially reducing computational overhead.

#### 2.4.4 DeepUMQA‐X for MassiveFold Model Selection

DeepUMQA‐X demonstrated superior performance in the selection of MassiveFold [1] models for hetero‐oligomeric complexes, where the method integrates structural clustering and modeling techniques to select the top 5 models. The MassiveFold model selection pipeline was implemented through a comprehensive five‐stage protocol: (1) Reference establishment: an initial model was randomly selected as a reference model, and all remaining models were structurally aligned against it using USalign [19] to compute structural similarity scores; (2) Structural clustering: models were grouped through hierarchical clustering based on their similarity scores, with a stringent cutoff threshold of TM‐score < 0.001 to maximize structural diversity; (3) Initial model pool construction: from each resulting cluster, a representative model was selected through random sampling to construct the primary model pool; (4) Model pool enhancement: to further diversify and improve model quality, we integrated additional high‐quality prediction models from multiple sources, including AlphaFold‐Multimer [50], AlphaFold3 [51], HDock [52], and our in‐house modeling methods (detailed in CASP16 abstract); (5) Final model selection: all candidate models underwent rigorous quality assessment using DeepUMQA‐X, from which the top five top‐ranked models were selected based on their predicted accuracy score.

#### 2.4.5 Atomic pLDDT Values in Predictions

Our group submitted structures with per‐atom accuracy self‐assessment derived from AF3, which were selected considering structure modeling methods and our quality assessment methods. Specifically, we generate many models using AlphaFold‐Multimer, AlphaFold3, HDock, and our in‐house modeling methods (see CASP16 abstract). For these models, top structures were selected using DeepUMQA‐X. If the final top five model was from AF3, the submitted structure remained unmodified.

DeepUMQA‐X is freely available at http://zhanglab‐bioinf.com/DeepUMQA‐X.

#### 2.4.6 Guijun Group Summary

What went right? DeepUMQA‐X demonstrated advanced performance in scoring, ranking, selection, and self‐assessment of complex models. Notably, it achieved the best performance in the lDDT metric of local interface accuracy assessment. Figure 4B shows an example of DeepUMQA‐X assessing local interface accuracy for a model of target T1206. The predicted quality plDDT demonstrates remarkable agreement with the true lDDT distribution, where red indicates low‐quality regions and blue indicates high‐quality regions. Meanwhile, DeepUMQA‐X also achieved the best performance in T1259o and H1222 targets for MassiveFold model selection, as shown in Figure 4C. For these two targets, DeepUMQA‐X selected top‐ranked structures highly similar to the native structure, with a penalty value of 0. According to the description of our EMA methods in the CASP16 abstract, we categorized different method types into consensus methods, quasi‐single model methods, and single‐model methods. The results indicate that our single‐model methods performed well in the estimation of global, interface, and local accuracy for complexes.

What went wrong? When evaluating global accuracy metrics for protein complexes, DeepUMQA‐X showed limited correlation performance, as evidenced by relatively low Pearson and Spearman correlation coefficients between its predicted quality scores and reference values. This correlation deficiency was particularly pronounced in the Oligo‐GDTTS [33] metric assessment, as illustrated in Figure 4D (left panel). Comprehensive analysis across all CASP16 targets revealed that the official consensus baseline method (AC) demonstrated the best performance, achieving Spearman (0.508) and Pearson (0.761) correlation coefficients. In comparison, DeepUMQA‐X showed relatively lower correlation values (Spearman = 0.376; Pearson = 0.643). However, in local interface accuracy assessment, DeepUMQA‐X's plDDT predictions exhibited significantly better performance than PatchDock, PatchQS [33] and CAD [28], as evidenced by higher Pearson correlation coefficients (Figure 4D, right panel). This performance discrepancy may stem from our method's reliance on lDDT and TM‐score metrics, potentially introducing assessment bias and limitations by neglecting other relevant quality evaluation metrics. Additionally, DeepUMQA‐X performs relatively poorly in the monomer MassiveFold model selection, as shown in Figure 4E for its results on monomer targets T1207 and T1226. For these two targets, the top‐ranked structure selected by DeepUMQA‐X had relatively low quality, with penalty values of 0.532 (T1207) and 0.529 (T1226). Similarly, this performance limitation primarily stems from our method's original design focus on complex model accuracy assessment, lacking specific optimization parameters and features tailored for monomer structure evaluation.

Assessors raised the question of whether we had any insight into why interface accuracy estimation would be better for GuijunLab‐QA than for GuijunLab‐Human. The GuijunLab‐QA and GuijunLab‐Human methods are both based on DeepUMQA‐X, integrating two single‐model evaluations and a consensus evaluation strategy, while relying on different high‐quality candidate model pools. Briefly, the main difference between the GuijunLab‐QA and GuijunLab‐Human methods lies in the criteria of model pool selection, where they filter the top 25% (GuijunLab‐QA) or 50% (GuijunLab‐Human) of high‐quality models from all candidate models based on pTM‐score and iplDDT. In fact, compared to the GuijunLab‐Human method that uses the top 50% parameter settings, the GuijunLab‐QA method applies the top 25% criterion resulting in a higher‐quality model pool, which enables it to improve the accuracy of the evaluation as reference models for structural consensus.

What did we learn? Through comprehensive analysis of complex structure prediction and estimation of model accuracy results from CASP16, we have gained critical insights into EMA track performance and identified multiple promising research directions for our group.

First, the blind assessment results demonstrated that consensus‐based methods still significantly outperform single‐model methods, while quasi‐single‐model methods exhibit outstanding performance. Since both consensus‐based and quasi‐single‐model methods rely on the accuracy of structure prediction methods, we hypothesize that advancements in model quality assessment may be lagging behind progress in structure prediction. Therefore, incorporating relevant information from structure prediction or adopting self‐assessment mechanisms similar to AlphaFold's evaluation framework could potentially enhance the performance of EMA methods.

Second, in the local accuracy estimation track, we observed that the Group MQA, which ranked first in local interface residue identification, significantly outperformed lower‐ranked methods, including those from GuijunLab. However, it exhibited poor performance in local interface accuracy assessment. To further investigate this discrepancy, we analyzed the true local quality performance in local interface residue identification, obtaining ROC AUC values of lDDT = 0.613, CAD = 0.584, PatchQS = 0.814, PatchDockQ = 0.817. These empirical findings strongly suggest that relying exclusively on local quality metrics may be inadequate for comprehensive evaluation across both local interface accuracy assessment and residue identification tasks.

Finally, in light of the newly established assessment tracks in structure prediction and model accuracy estimation, we believe that several critical research directions are essential for advancing model quality assessment methodologies: (1) development of robust evaluation frameworks for structures with unknown stoichiometry and dynamic conformational states, (2) implementation of per‐atom accuracy assessment protocols, and (3) establishment of efficient massive model selection strategies. These advancements are expected to significantly contribute to the progress of structural biology by providing more comprehensive and accurate tools for protein structure analysis.

### 2.5 Shortle Group Methods

The native state of a protein is presumed to be the conformation of lowest free energy. Although this free energy cannot be calculated, it can be approximated by comparing structural details of a predicted model with the statistics of the same features in high‐resolution x‐ray structures. To the extent that details of structure are the consequence of their low free energy, as assumed by the Boltzmann hypothesis, comparison of numerous diverse structural features with the statistics of their occurrence in real proteins should provide a rough estimate of the overall free energy. Here we apply this idea to assessing the large sets of MassiveFold models available in CASP16, using several structural details that quantify atom‐atom overlap, Ramachandran propensities, hydrogen bonds, and atom‐atom interactions. Parameter values were calculated, ranked by placement into 20 equal value bins, and then added to give a final score of overall model quality. The results of this approach were significantly better than the average of the AI‐based methods and suggest improved quality assessment could be achieved by comparing more parameters, by using more appropriate high‐resolution x‐ray structure libraries, and by utilizing more rigorous methods of balancing the contribution of individual parameters to the final score.

In the context of physical chemistry, low global energy is achieved by optimizing most or all of the different bonding interactions and structural arrangements of atoms to near their individual lowest free energies. Unfortunately, at present, a sufficiently quantitative understanding of the forces involved to allow these minima to be calculated is not available. The best one can do is approximate parts of a protein's free energy function by examining high‐resolution x‐ray structures and identifying those local atom arrangements that differ in quantifiable detail from those found in lower accuracy structures. Presumably, such common arrangements of atoms represent structures that achieve low free energies. Stated in more physical chemical terms, if the Boltzmann approximation holds [53], then statistical parameters derived from an ensemble of highly accurate structures will reflect the potential of mean force for components of the overall free energy.

The methods employed in this work use a conventional strategy [54, 55]: analyze the statistics of structure descriptors of atom groupings that consistently correlate with high experimental accuracy; in other words, those features that are more common at higher resolution than in lower resolution, as defined by the experimentalist as the model's “x‐ray resolution.” With the larger number of data points collected by x‐ray crystallography required to achieve higher resolution, the crystallographer's three‐dimensional model is confined to a smaller and smaller set of conformational possibilities that fit the data. The same argument can be made for the correspondence of the two experimental R‐factors with model accuracy.

Identifying those structural details that most consistently correlate with high experimental resolution requires considerable trial and error. In this work, the statistics of different measures of atom‐atom overlap, backbone phi/psi and chi1 angles, hydrogen bonds, and atom‐atom distance distributions have been calculated from a set of +2700 monomeric PDB x‐ray structures with reported resolutions of 1.4A or less.

Obviously, the choice and combination of parameters reported here is based on the author's experience and intuition. In this work, various parameters developed by the author over the past 12 rounds of CASP were used. No attempts were made to refine/adjust the methods of their calculation to reduce or eliminate correlations among them, or to combine them in ways other than simple addition. Each parameter's performance was evaluated by comparing the correlation of calculated values versus x‐ray resolution and also by success in the discrimination of the correct structure challenged by sets of 1000 decoy models.

**FIGURE 5:**
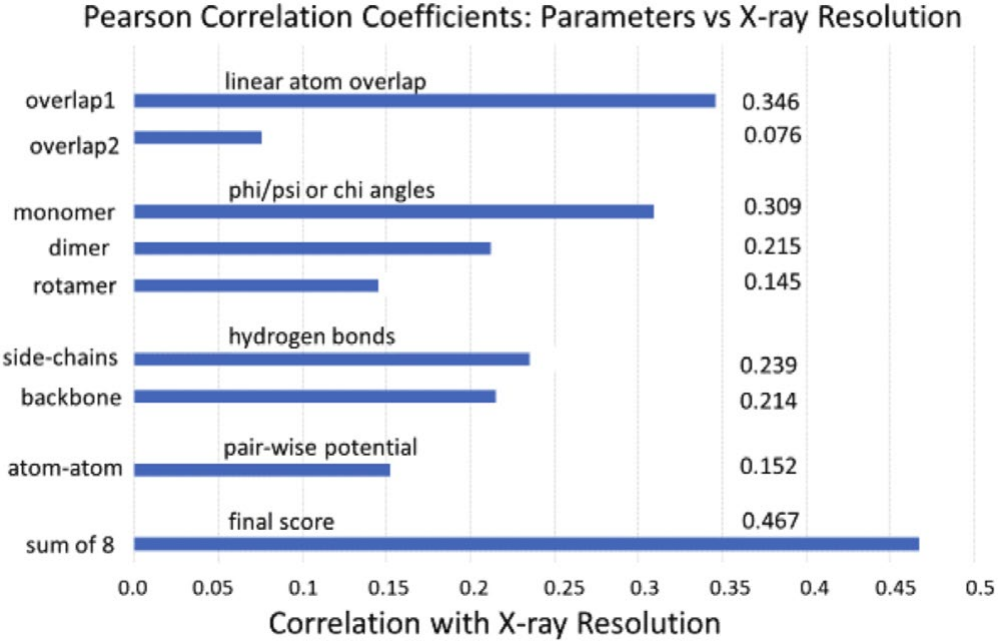
Statistical correlations of the eight scoring parameters used in this work versus the x‐ray crystallographers' measure of resolution; that is, these correlation coefficients serve as rough measures of a parameter's efficacy in assessing the accuracy of a structure.

Figure 5 shows a list of the eight parameters used in CASP16 for quality assessment (QA3), plus the results of applying them to a set of 1800 proteins from the PDB with reported x‐ray resolutions from 0.7A to 2.5A. The parameter values were rank ordered from lowest to highest energy and then divided into 20 bins with an equal number of values per bin.

Atom‐atom overlap was not raised to a higher power, as is done for energy calculations. Rather, a linear measure of overlap versus structural error seems more reasonable. The first of the two parameters shown in Figure 5, the linear overlap between immediately adjacent residues i to i + 1 (overlap1), correlates with x‐ray resolution more strongly than overlap2, which is total overlap calculated between residues separated by 5 or more. Surprisingly, local overlap1 displayed the highest correlation with x‐ray resolution of any of the other 7 parameters.

Next in the list on Figure 5 are three statistical propensities for backbone phi/psi and chi1 angles [56, 57]. To calculate the monomer potential, the Ramachandran plot of phi/psi angles for single residues was divided into 137 bins, with calculation of this parameter for each amino acid type and separately for alpha helices, beta strands, and turn/loop/irregular segments [56]. Notably, the monomer parameter shows the second highest correlation with x‐ray resolution. For the dimer potential, phi/psi angles for each of two adjacent residues were divided into 26 bins. Only residue pairs for beta strands and for junctions between helix/strand and irregular segments were calculated, and these two values combined form the dimer potential. The rotamer potential was calculated for single amino acid residues in each of the 3 separate secondary structural types, with the phi/psi angles assigned to 26 bins and the x1 angle to 3 bins.

Hydrogen bonds for side‐chain to side‐chain or backbone atoms consisted of a distance‐dependent statistical potential of nine hydrogen bond donor atoms and five hydrogen bond acceptors. For backbone to backbone hydrogen bonds, the potential developed by the Baker lab was employed [58].

The atom‐atom interaction parameter is based on 86 atom types and was scored over a surface‐to‐surface distance range of 0 (contact) to 2.0 Å divided into 0.1 Å bins.

While the individual correlation coefficients shown in Figure 5 are not large and several may not be statistically significant, when the ranked values are simply added together for the final_score, the correlation coefficient is 0.467. If the correlation is made between final_score and the R‐factor or the R‐free factor instead of the x‐ray resolution, the results are essentially unchanged (not shown). The relationship between these three experimental values and the accuracy relative to the “true” structure cannot be clearly defined, but they do represent an approximate measure of the range of conformations that could fit the experimental data.

The original plan to score the 8000 models generated by the Massive protocol was to convert each value calculated for a model's parameter value into a ranking relative to the scores of the best PDB structures as mentioned above. However, for all of the targets, only a small number of parameters for a very small number of these models scored at or lower than the highest energy bin. Consequently, instead, the 8000 model scores for each parameter were rank ordered into 20 equal bins, and the bin numbers for the 8 parameters in Figure 5 were simply added together to give the final score, using this value as the measure of quality.

The performance rating of final_score in the QA3 challenge given by the assessor was as follows: For monomeric targets, the 5 structures deemed highest in quality for each target were tied for 3rd place among the 22 different contributing groups. For the homo‐oligomeric targets, the submitted structures were ranked 5 in accuracy compared to 24 groups, and for the hetero‐oligomeric targets, ranking was 11/24.

#### 2.5.1 Shortle Group Summary

These initial results establish that, for the assessment of model accuracy, there is merit in the use of statistics derived from the frequencies of multiple different structural details as proxies for components of a model's free energy. Furthermore, these results suggest that this approach could be greatly improved by making the following modifications, which were not applied in CASP16:

1. The final scoring function should use Multiple Regression to generate a function that combines individual parameter values to reduce correlations among parameters and to properly weight each parameter.
2. Separate probability tables for each parameter should be calculated from PDB structures for three distinct libraries: monomeric proteins, homo‐oligomers, and hetero‐oligomers. In this work, only one library of monomeric proteins was used for all probability tables.
3. For oligomeric proteins, better still would be separate tables for interfacial residues versus residues outside the interface.

## Author Contributions

**Alisia Fadini:** investigation, writing – original draft, writing – review and editing. **Recep Adiyaman:** investigation, writing – review and editing, funding acquisition. **Shaima N. Alhaddad:** investigation, writing – review and editing, funding acquisition. **Behnosh Behzadi:** investigation, writing – review and editing. **Jianlin Cheng:** funding acquisition, conceptualization, investigation, writing – review and editing, writing – original draft. **Xinyue Cui:** investigation, writing – review and editing. **Nicholas S. Edmunds:** investigation, writing – review and editing. **Lydia Freddolino:** funding acquisition, conceptualization, investigation, writing – review and editing, writing – original draft. **Ahmet G. Genc:** funding acquisition, investigation, writing – review and editing. **Fang Liang:** investigation, writing – review and editing. **Dong Liu:** investigation, writing – review and editing. **Jian Liu:** investigation, writing – review and editing. **Quancheng Liu:** investigation, writing – review and editing. **Liam J. McGuffin:** funding acquisition, conceptualization, investigation, writing – review and editing, writing – original draft. **Pawan Neupane:** investigation, writing – review and editing. **Chunxiang Peng:** investigation, writing – review and editing. **David R. Shortle:** funding acquisition, conceptualization, investigation, writing – review and editing, writing – original draft. **Meng Sun:** investigation, writing – review and editing. **Haodong Wang:** investigation, writing – review and editing. **Qiqige Wuyun:** investigation, writing – review and editing. **Guijun Zhang:** funding acquisition. **Xuanfeng Zhao:** investigation, writing – review and editing. **Wei Zheng:** funding acquisition, conceptualization, investigation, writing – review and editing, writing – original draft. **Randy J. Read:** funding acquisition, conceptualization, investigation, writing – original draft.

## Conflicts of Interest

The authors declare no conflicts of interest.

## Data Availability

The data that support the findings of this study are openly available in CASP16 at https://predictioncenter.org/casp16.
